# Crural Neuralgia among Dentists

**Published:** 1882-10

**Authors:** 


### CRURAL NEURALGIA AMONG DENTISTS.

Dr. J. B. Sutton makes the following curious observation in the *Lancet:*

On June 20th I was called to a gentleman said to be suffering severely from sciatica. The patient was resting on his right side, complaining of intense pain in the left loin, radiating thence along the outer and anterior aspect of the left thigh whenever he attempted to move. Firm pressure, applied between tuberosity of ischium and great trochanter of femur was painless, but the instant one touched the skin immediately over the erector spinæ muscle severe pain was evoked, extending down the thigh to the knee-joint, mapping out exactly the course of the anterior, crural and external cutaneous nerves. Pressure over the points of exit of the second, third and fourth lumbar nerves from the spinal canal caused excessive pain. The nerves at fault were clearly the second, third and fourth lumbar, the hyperæsthetic area in the loin clearly corresponding to the distribution of the posterior divisions of these nerve trunks. The case was obviously not one of sciatica, but crural neuralgia, having a very unusual distribution ; the ordinary forms of this disease extend to the foot and toes of the affected leg. Belladonna and aconite were applied locally, strychnia internally ; the patient was convalescent in seven days.

Two days later a similar case came under my notice, and a third has been reported to me with exactly similar symptoms. Curiously enough, the three patients were dentists, engaged in the active duties of their profession. It appears exceedingly probable that this painful affection may be explained by the fact that when dentists operate they alway stand at the right side of the patient, consequently, when manipulating cavities in teeth difficult of access it is necessary to throw themselves into a constrained attitude, whereby the lumbar vertebræ are slightly flexed anteriorly, but flexed laterally to a considerable degree. It is necessary, sometimes, to maintain this cramped position for long periods, the temporary distortion being even more exaggerated when the dental engine is being used. These combined flexions cause the lumbar nerves to become congested, irritated, and possibly injuriously nipped as they pass through the intervertebral foramina, thus giving rise to the symptoms detailed above.

